# We’re talking about practice: examining the shifting role of FLS in surgical education

**DOI:** 10.1007/s00464-025-12414-9

**Published:** 2025-12-23

**Authors:** Gabrielle Falco, Alex Dunaway, Stephen Ranney, William Sweeney, Zachary Taylor, Christopher Dyke

**Affiliations:** 1 Department of General Surgery, Brooke Army Medical Center, Fort Sam Houston, TX USA; 2 Department of General Surgery, Naval Medical Center Portsmouth, Portsmouth, VA USA; 3 Department of General Surgery, Walter Reed National Military Medical Center, Bethesda, MD USA

**Keywords:** Fundamentals of laparoscopic surgery, Surgical education, Needs assessment, Simulation-based curriculum, Laparoscopic surgery training

## Abstract

**Background:**

In 2004, the Society of Gastrointestinal and Endoscopic Surgeons launched the fundamentals of laparoscopic surgery (FLS) program to ensure surgeons had the minimum knowledge, judgment, and technical skills to perform basic laparoscopic operations. Since FLS’s implementation, laparoscopic surgery rates have risen, and its role as a summative assessment has shifted as programs introduce FLS earlier in training. We hypothesize that surgical trainees only practice laparoscopy in a box trainer to complete FLS and seek to define the relationship of FLS and attitudes toward the independent practice of laparoscopy in general surgery trainees.

**Methods:**

A survey was distributed to three general surgery residency programs using five-point Likert, nominal, and ratio scales to collect data on independent laparoscopy practice, opinions on FLS skills, and its role in preparing for operative cases. Data analysis focused on response rates of answer choices.

**Results:**

Of the 88 residents polled, thirty-one residents responded to the survey (35%), of which 45.2% (14) have passed the FLS exam. The first-time pass rate was 100%. Approximately, one-third of residents who completed FLS state they regularly practiced laparoscopy before FLS completion (28.6% agree, 7.1% strongly agree). That number decreased to 7.1% after FLS completion (7.1% agree, 0% strongly agree). About half of residents only practice skills from the FLS skills test (19.4% strongly agree, 29.0% agree). Yet, only a third (32.3%) agreed that FLS skills are transferable to complex laparoscopy cases.

**Conclusions:**

Simulated laparoscopic practice decreases after completion of FLS, and the majority of residents only engage in practice using FLS skills. FLS has become the main driver of independent laparoscopic practice for many residents. An emphasis on self-directed learning and expanded laparoscopic skills taught in simulation could increase time spent practicing and more easily transfer skills to the operating room.

Laparoscopic surgery has become a cornerstone of modern surgical practice, particularly over the last decade as it has evolved into the standard of care for many abdominal procedures. Advancements in technology, greater surgeon comfort, and improved patient outcomes such as reduced postoperative pain, shorter hospital stays, and faster return to normal activities have driven its widespread adoption [1–5]. With this rise in laparoscopic surgery, the Society of American Gastrointestinal and Endoscopic Surgeons (SAGES) and the American College of Surgeons (ACS) developed and launched the Fundamentals of Laparoscopic Surgery (FLS) program in 2004, to address concerns about the variability of training in laparoscopic surgery.

While the FLS program was initially introduced as a summative examination, its role has evolved into a foundational training tool. From 2004 to 2009, only 12% of residents who took FLS were junior residents [6]. By 2020, a survey conducted by Schmeiderer et al. demonstrated that 33.09% of junior residents had completed FLS [7]. Despite no universally accepted changes, the role of FLS in surgical residency education has shifted from a summative safety exam to an introductory education tool.

Since the implementation of FLS, the rates of laparoscopic surgery have risen. A review completed by Bingmer et al. showed that the mean number of laparoscopic cases performed by general surgery residents increased from 23.6 in 1999–2000 to 135.6 (462%) in 2017–2018 [8]. With this large increase in operative laparoscopic training, many general surgery staff and residents are questioning the current utility and future direction of the FLS program. Research also suggests that FLS may not fully prepare residents for more advanced laparoscopic procedures, as many residents continue to require additional hands-on training before achieving operative autonomy [9]. Additionally, while FLS ensures competency in basic laparoscopic skills, there is ongoing debate regarding whether the program should expand to include more complex skills, such as laparoscopic suturing and advanced dissection techniques [10]. This study seeks to define the relationship of FLS and attitudes toward the independent practice of laparoscopy in general surgery trainees.

## Materials and methods

We conducted a cross-sectional survey study across three general surgery residency programs. The study was approved as exempt research by the institutional review board (Reference # 981447). The survey was developed by the study authors and included 19 items using a combination of five-point Likert-type, nominal, and ratio scale questions. Survey domains included the frequency and duration of independent laparoscopic practice before and after FLS certification, attitudes regarding the utility and transferability of FLS skills, types of skills practiced, and use of FLS training in preparation for operative cases.

To ensure clarity and relevance of the survey questions, the initial version was piloted with one urology and one obstetrics and gynecology (OB/GYN) resident, both of whom had prior experience with laparoscopic surgery and FLS. These two residents were chosen specifically because they were familiar with laparoscopic training and were not part of the targeted population of general surgery residents. Based on their feedback, edits were made to improve the wording and structure of several questions.

The finalized survey was distributed electronically via email and text using an anonymous Google Forms link. Authors from each institution assisted in disseminating the survey to all categorical general surgery residents. Participation was voluntary, and no incentives were provided. The survey remained open for a period of two weeks, with two reminder notifications sent during that time.

Survey responses were collected and stored within the Google program. Data analysis was performed using Google Sheets. Descriptive statistics were used to summarize resident demographics, frequency of practice, and Likert-scale responses. Categorical variables were analyzed using frequencies and percentages. No inferential statistical testing was performed given the small sample size and exploratory nature of the study.

Responses to open-ended survey questions were reviewed by the study team. Due to the small number of qualitative responses, formal thematic analysis was not performed. Instead, comments were analyzed descriptively and grouped based on how they aligned with the quantitative findings, particularly regarding attitudes toward FLS content, simulation access, and coaching availability.

A full copy of the survey instrument used in this study is provided in Appendix A.

## Results

### Quantitative insights

**Fig. 1:**
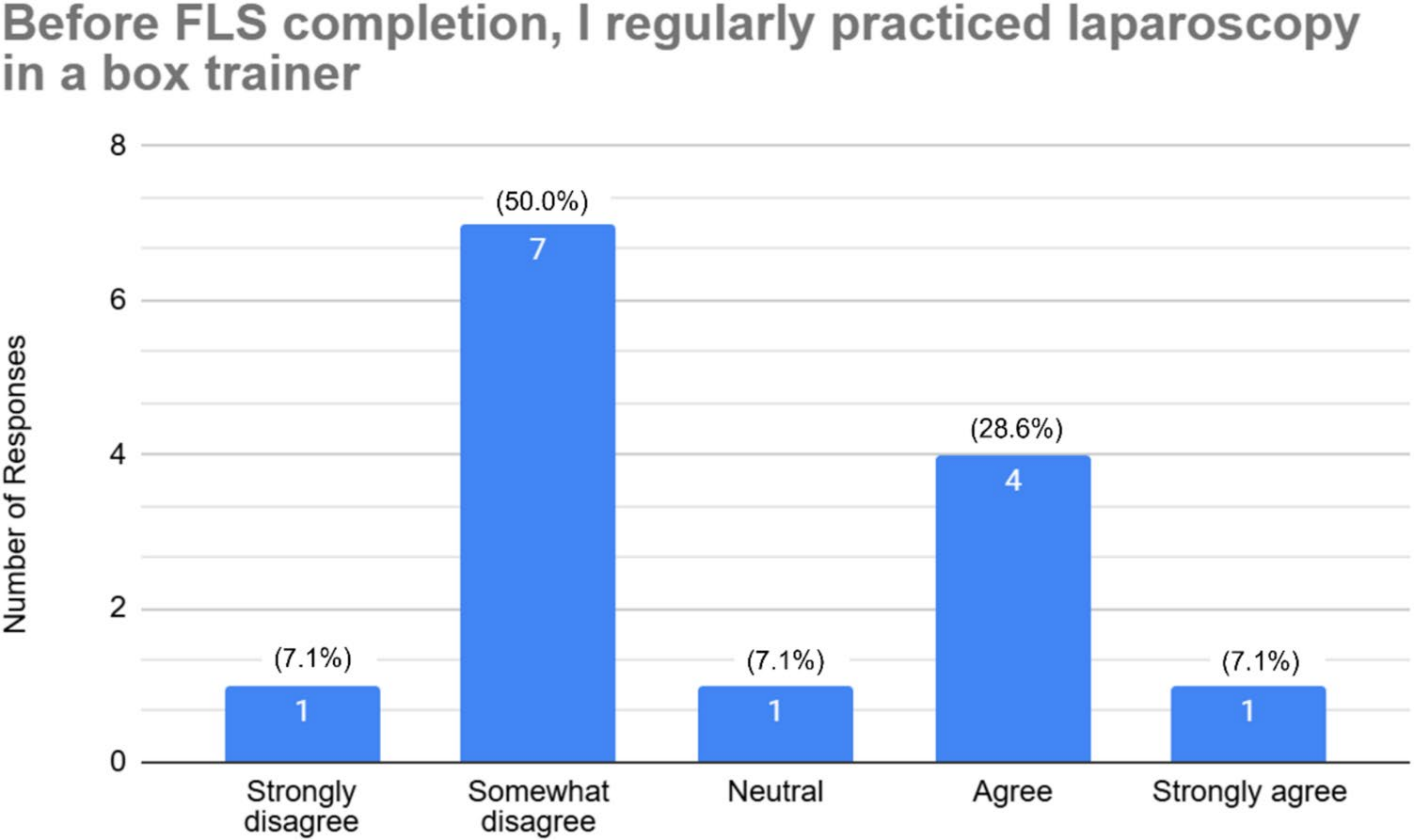
Resident responses to laparoscopic practice before FLS completion

**Fig. 2:**
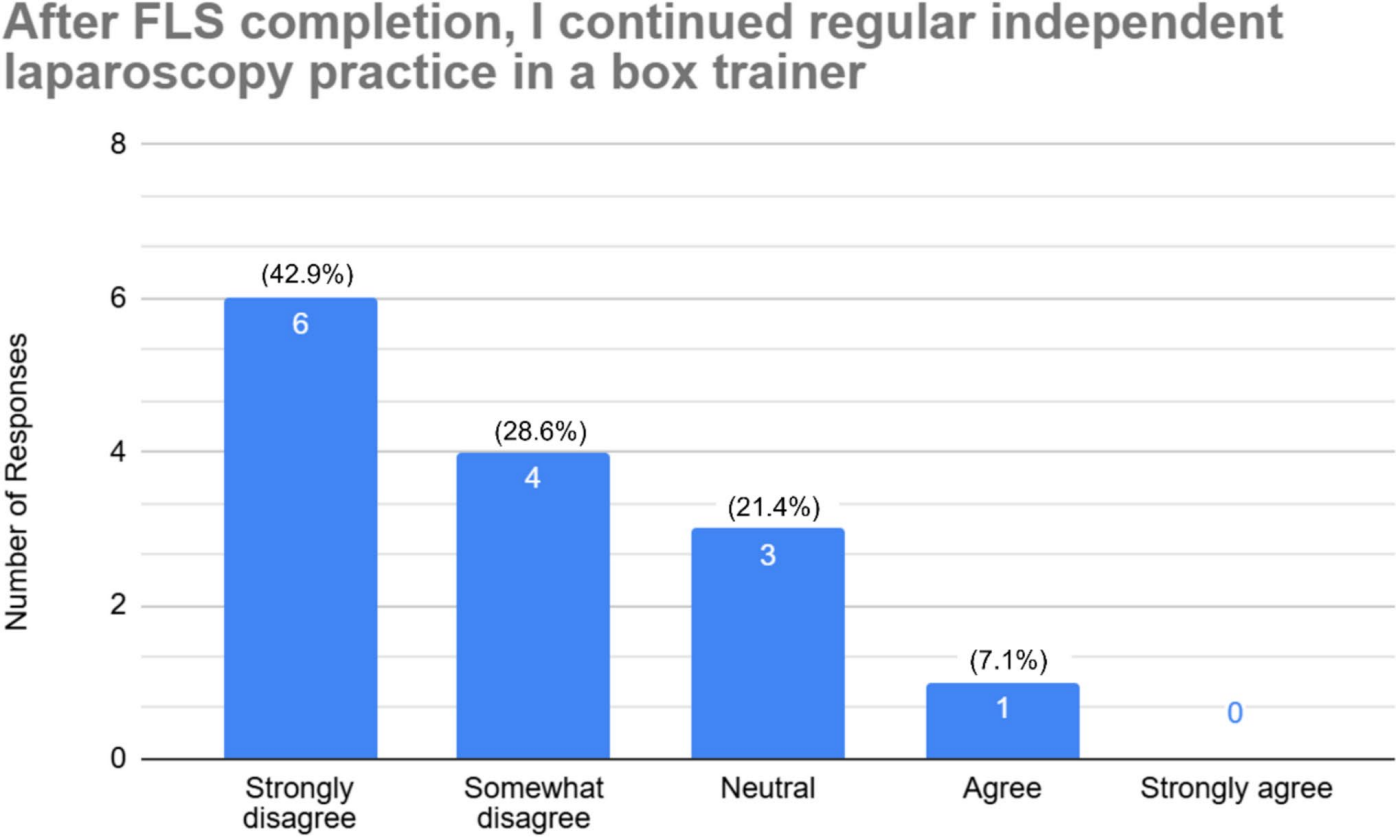
Resident responses to laparoscopic practice after FLS completion

The survey was sent to a total of 88 general surgery residents, and 31 responded to the survey (~ 35% response rate). The cohort included PGY-1 through PGY-5 residents (residents in non-clinical research years were also included). 45.2% (*n* = 14) reported having completed the FLS examination. Regarding the timing of FLS completion, all residents who completed the exam did so in PGY-2, PGY-3, or research year. The first-time pass rate was 100%. Among those who completed FLS, 28.6% (*n* = 4) agreed and 7.1% (*n* = 1) strongly agreed that they regularly practiced independently prior to the exam, seen in Fig. 1. In contrast, only 7.2% (*n* = 1) reported continued regular practice after FLS completion, with the majority selecting neutral (21.4%, *n* = 3) or disagreeing (71.4%, *n* = 10) responses, shown in Fig. 2.

**Fig. 3:**
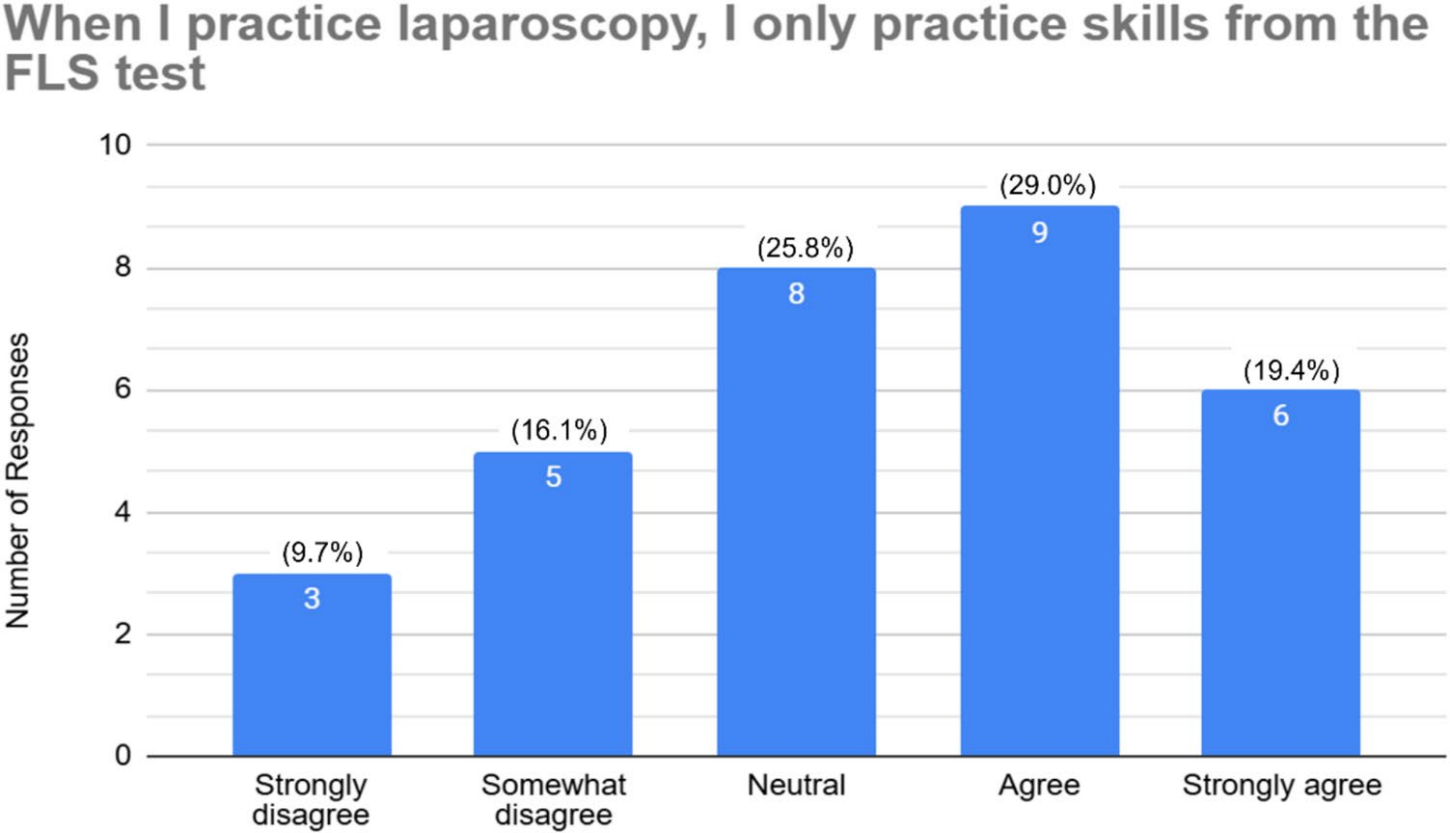
Resident responses to only practicing the FLS skills on the box trainer

**Fig. 4:**
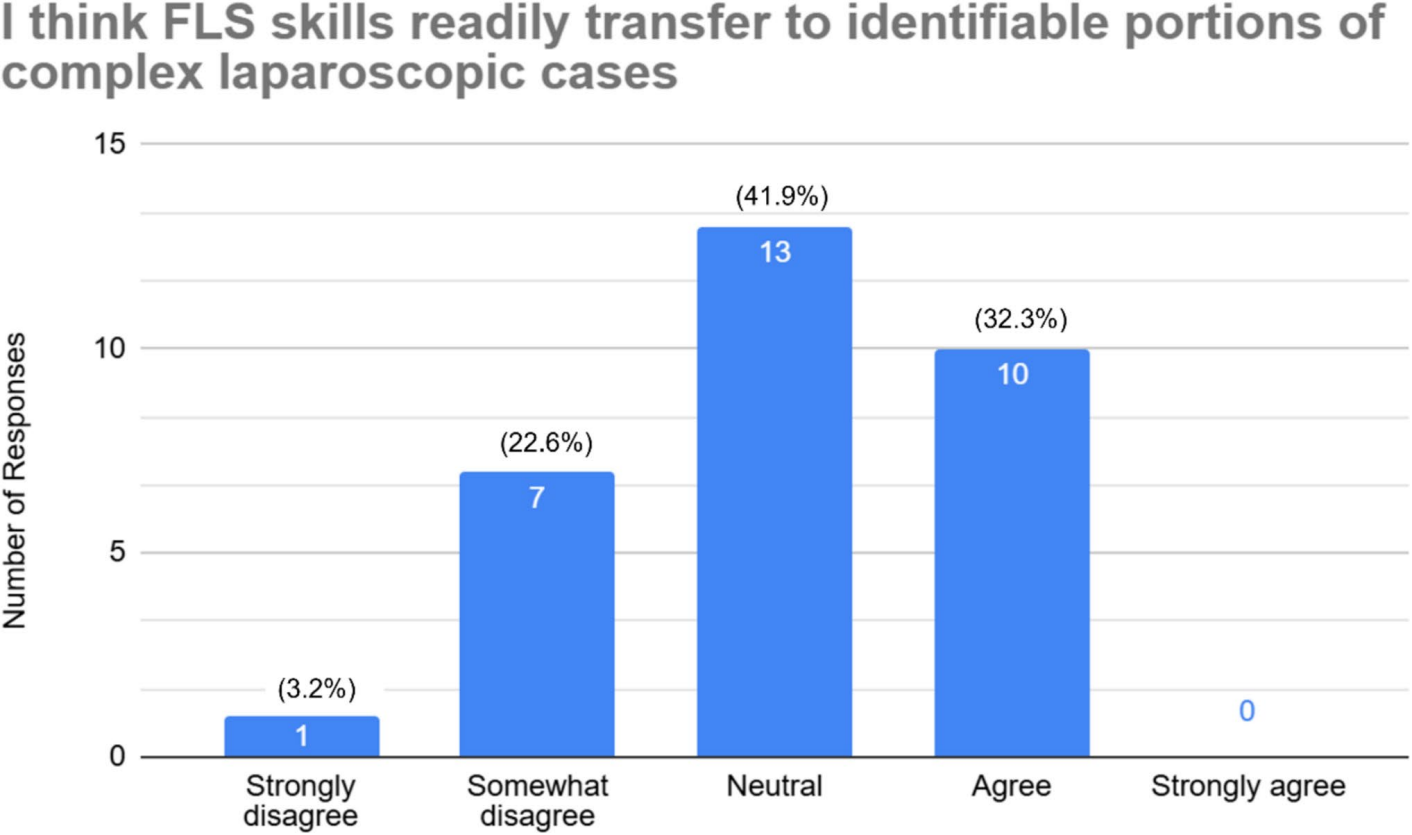
Resident thoughts on the transferability of FLS skills to laparoscopic cases

Regarding content of their practice, 48.4% (*n* = 15) agreed or strongly agreed that they practiced only the five core FLS skills (peg transfer, pattern cutting, loop ligation, extracorporeal suturing, and intracorporeal suturing), while 25.8% (*n* = 8) reported disagreeing or strongly disagreeing, indicating some residents are practicing additional skills. Figure 3 shows the responses regarding practice using only the FLS skills. In terms of perceived applicability, 32.3% (*n* = 10) agreed that FLS skills were transferable to complex laparoscopic cases, while 41.9% (*n* = 13) responded neutrally and 25.8% (*n* = 8) expressed some form of disagreement, seen in Fig. 4. Additionally, just 10% (*n* = 3) of residents indicated they practiced independently in preparation for complex operative cases.

When analyzed by postgraduate year, most residents reported practicing 1–3 times per year on a laparoscopic trainer during postgraduate years 1 and 2. Coaching or instruction from faculty outside of the operating room was rare, with the majority of respondents reporting 0–3 coached sessions annually across all years of training.

**Fig. 5:**
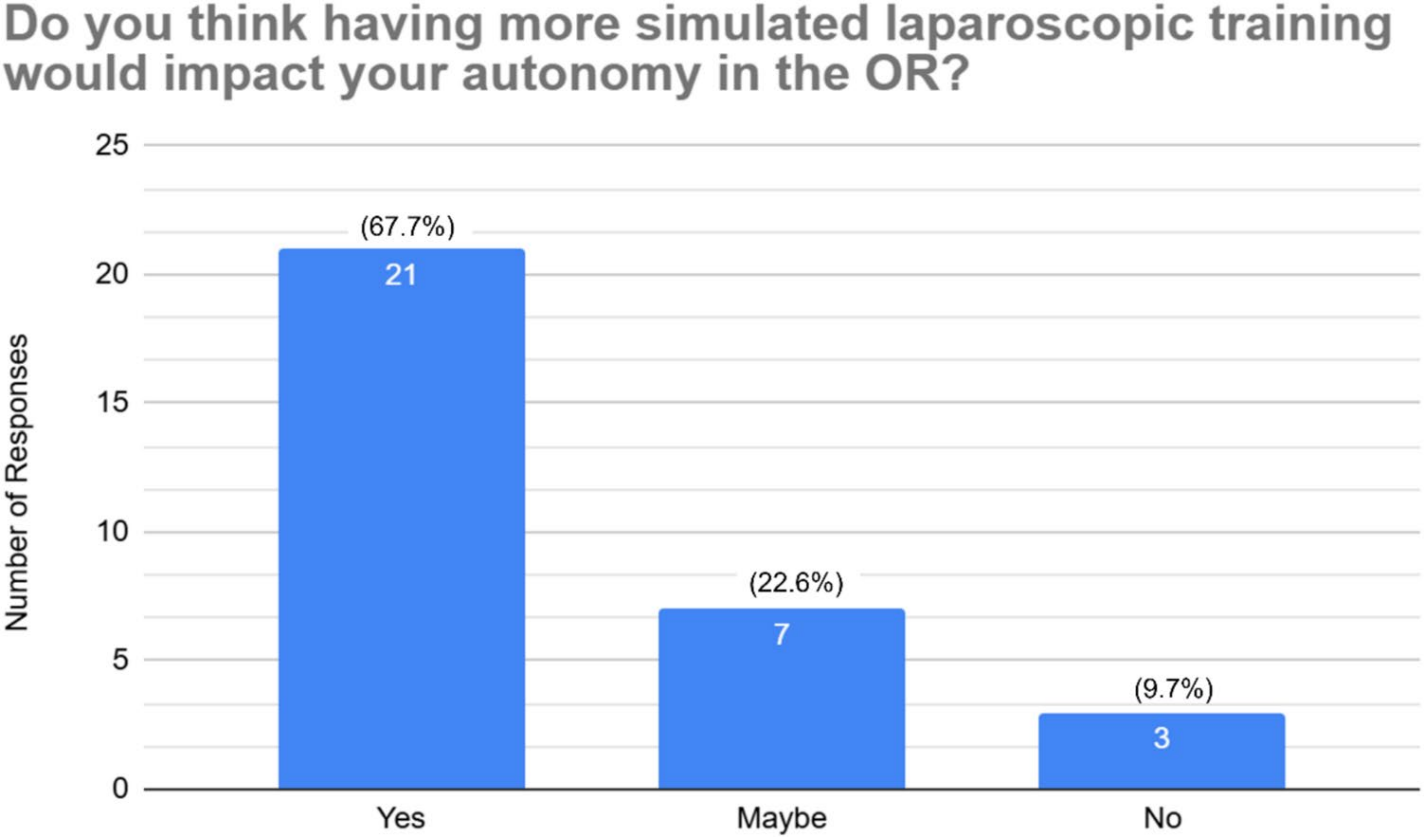
Resident thoughts on if simulated laparoscopic training will impact autonomy in the operating room

When asked about the value of additional simulation, 90.3% (*n* = 28) of respondents reported that more simulated training would have a positive impact on their operative autonomy; 67.7% (*n* = 21) responded “Yes,” while 22.6% (*n* = 7) responded “Maybe.” Fig. 5 shows the complete breakdown of these results.

### Qualitative insights

Open-ended responses provided additional depth and context to the quantitative findings. Several residents felt that FLS was overly simplistic or limited in scope. One respondent stated,

FLS skills are too basic. Probably fine for the test to avoid the test becoming an onerous requirement but not complex to simulate important skills like understanding how to use two hands... or identifying and exploiting areas of tension.

Another respondent echoed this sentiment.

There are several laparoscopic skills that could be practiced in a trainer outside of the OR that could improve preparation... E.g., suturing from different angles, advanced knot tying, diagnostic laparoscopy.

Others emphasized the need for expanded training:

I think additional tasks do need to be developed, separate from the ones that are on the FLS test. For example, variations to laparoscopic suturing would be helpful!

A common theme was the lack of structured coaching and supervised instruction.

More supervised teaching for suturing laparoscopically.

More practice with an expert for error recognition in the Sim lab would be beneficial.

Barriers to accessing training were also noted.

Wish for more guidance with the machines early in training.

I think showing the interns where the sim lab is and what the FLS tested skills are would be helpful—I found it by accident.

Some respondents expressed a preference for operative-based learning over simulation.

The best place to learn laparoscopic skills is in the OR.

## Discussion

Simulation has been proven to be safe and effective for residents to acquire laparoscopic surgical skills before entering the operating room [11]. Prior simulation practice or laparoscopic training certification significantly improves performance, reflected by achieving higher scores and passing FLS proficiency scores [12]. This underscores the value of FLS as a simulation-based program designed to ensure that all surgeons possess the minimum knowledge, judgment, and technical skill required for safe laparoscopy but additionally to help residents build core laparoscopic skills prior to operating on patients.

Our findings suggest that while the FLS program plays a crucial role in establishing fundamental laparoscopic skills, it has become the primary driver, and for many, the endpoint, of independent laparoscopic practice. Most residents who completed FLS reported limited continued engagement with box trainer simulation thereafter. Only 7.1% of FLS-certified residents reported regularly practicing after the exam, compared to over one-third who did so beforehand. This decline in self-directed practice following FLS completion raises concerns regarding skill retention and readiness for advanced operative tasks.

Higgins et al. found that FLS certification led to improved resident performance and increased autonomy in laparoscopic cases [13]. Similarly, Cullinan et al. advocated for earlier FLS testing, suggesting that it may improve skill acquisition before residents take on more complex laparoscopic cases [9]. Our data reflect this shift, with most residents completing the exam in postgraduate years 2 and 3. However, our results also indicate that early testing may inadvertently signal the end of simulation-based practice rather than serve as a springboard for continued skills development.

**Fig. 6:**
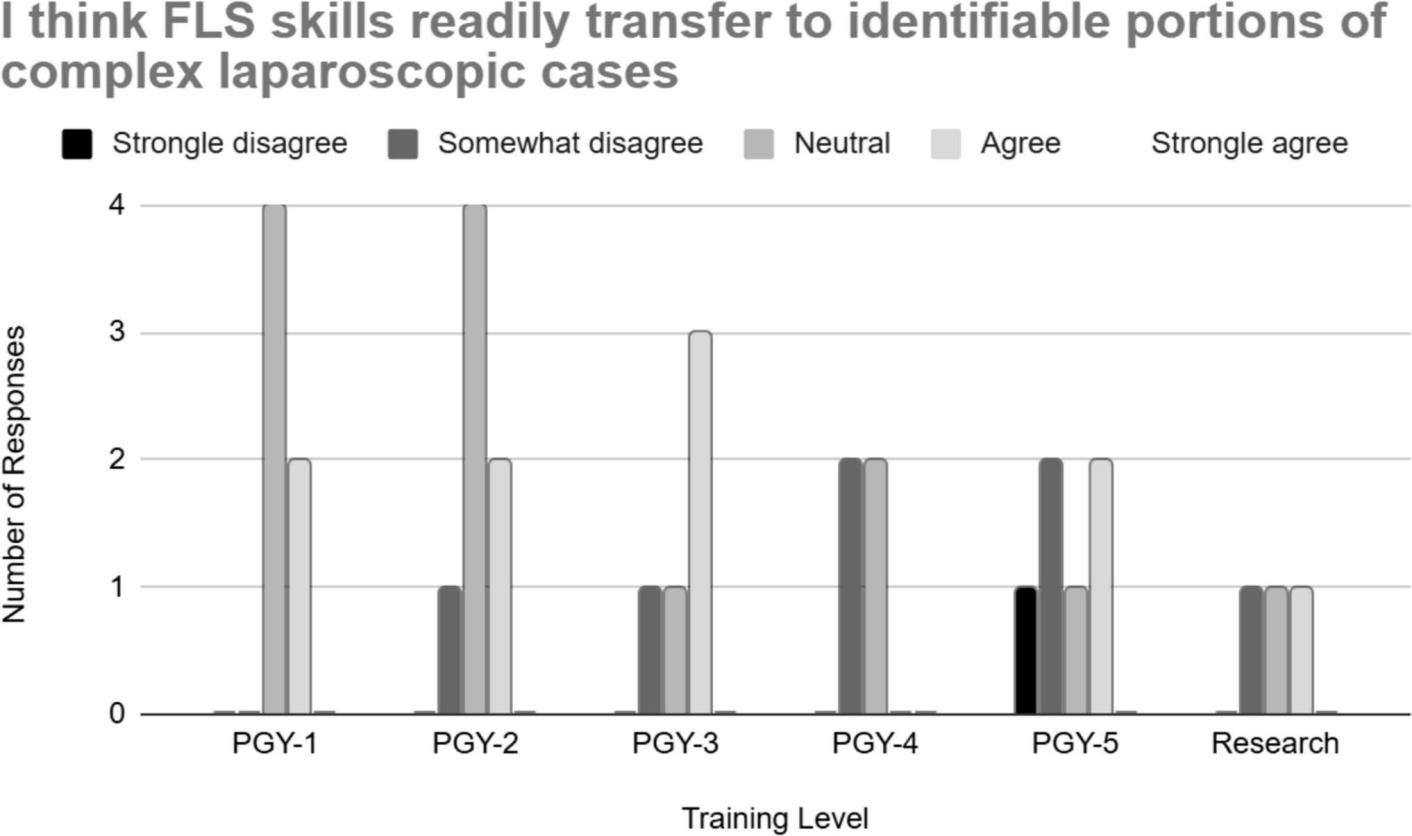
Resident thoughts on the transferability of FLS skills to laparoscopic cases by training level

Despite the benefits of FLS, some studies have questioned whether it is sufficient for ensuring proficiency in complex laparoscopic procedures. Hafford et al. emphasized that while FLS improves technical competency, additional structured simulation and intraoperative experience are essential to translating these skills into real-world laparoscopic performance [10]. Our study supports this concern: only one-third of respondents believed FLS skills were transferable to complex laparoscopic operations, and just 10% reported practicing in preparation for complex cases. When further breaking down responses regarding skill transferability by level of training, there is a trend toward less positive views for FLS skill transferability to complex laparoscopic cases as residents advance in training. Figure 6 shows that no PGY4 and only 2 PGY5 responders agreed that these skills readily transferred to more complex cases, while more PGY1 through PGY3 responders agreed or were neutral. Given the purpose for which FLS was created, attitudes toward skill transferability to complex laparoscopy are more an indictment on the failure to establish broadly scaled curricula that move beyond fundamental technical skills. This is further reflected in responses showing that almost half (48.4%) of responders only practice tasks included in the FLS skills examination, while only one quarter (25.8%) disagreed with this statement inferring that they practiced other skills. Of the few curricula developed to teach advanced laparoscopic skills, the most notable is the Advanced Training in Laparoscopic Suturing (ATLAS) program launched by the Association for Surgical Education (ASE) in 2022 [14]. While this program has the validity evidence to support broad-scale adoption, it is not taught at any of the programs included in this survey and its adoption among training programs has not been quantified. This is likely due to a variety of reasons common to all simulation-based practice. Among these are attitudes and desire to participate in independent simulation-based practice, awareness of these curricula, competition for resources, and cost and availability of simulation task trainers.

The qualitative feedback further emphasized these limitations. Several residents described the FLS tasks as overly simplistic, noting that the skills do not simulate the cognitive demands of operative laparoscopy, such as managing spatial orientation, retraction, or complex suturing. Others expressed a desire for training modules that incorporate more advanced or variable techniques, such as suturing from different angles, port placement strategies, or bimanual dissection, that better mirror real operative challenges.

Additionally, many respondents noted the lack of structured coaching and limited guidance in using simulation equipment. While some residents reported practicing 1–3 times per year during PGY-1 and PGY-2, practice rates declined substantially after PGY-3, and most reported receiving 0–3 coached sessions annually across all levels. These findings highlight a gap in faculty engagement and formalized simulation instruction.

Encouragingly, nearly all residents (90.3%) believe that more simulation would improve their operative autonomy. Bingmer et al. (2020) documented a 462% increase in laparoscopic case volumes among general surgery residents between 1999 and 2018, reinforcing the need for laparoscopic training programs, like FLS, to evolve alongside rising case complexity and volume [8]. Programs may also consider incentivizing continued laparoscopic skill development beyond FLS certification, either through longitudinal curricula, simulation milestones, or embedded feedback mechanisms.

### Limitations

This study has several limitations that should be considered when interpreting the results. First, the sample size was small including only three geographically distinct programs, and the survey did not undergo rigorous validation and testing. While the response rate of approximately 35% is consistent with similar survey-based studies, the limited number of participants may affect the generalizability of the findings to broader surgical training populations. The survey used in this study was developed in-house and underwent informal review by urology and OB/GYN trainees to improve clarity and phrasing. However, it did not undergo formal psychometric validation or pilot testing. As such, the reliability and validity of the instrument have not been established, and findings are subject to measurement bias. Additionally affecting the generalizability, all three of these programs are military health system-affiliated general surgery programs and may not reflect the population of non-military programs. While military residency programs depend on partnerships with civilian programs for surgical training, there are some distinct differences. There is a transient nature of staff surgeons at military treatment facilities as they face frequent deployments and changes of duty station. As a result, there is a heightened awareness of skill decay leading to more emphasis on simulation or cadaver-based training offered in robust simulations labs at larger facilities. Residents, in turn, benefit from military specific simulation curriculum and generally have access to task trainers needed to prepare for Fundamentals exams. A more widely distributed survey across a larger variety of training programs could provide further insight that can be more easily generalized, regarding attitudes toward independent practice in laparoscopy and their relation to FLS.

Second, the study relied on self-reported data, which may be subject to recall bias or social desirability bias. Residents may have under- or overestimated their practice habits or attitudes toward FLS and simulation. Additionally, the data collected were cross-sectional and do not provide information on how individual attitudes or behaviors change over time.

Third, although the survey included both quantitative and qualitative items, the qualitative data were limited in depth due to the brief nature of open-ended responses. While these comments offered useful insights, more comprehensive qualitative methods such as interviews or focus groups could provide a richer understanding of residents’ perspectives on laparoscopic simulation training.

Lastly, this study focused on general surgery residents and excluded other surgical specialties that also utilize FLS. As such, the findings may not fully capture how trainees in fields such as gynecology or urology experience and value FLS, despite their comparable exposure to laparoscopic techniques.

### Future directions

This survey showed decreasing rates of independent, simulation-based practice after completion of the FLS examination and as trainees advance in PGY level. Most concerning is the paradoxical finding suggested by these data that as many as two thirds of residents did not practice regularly even before FLS certification even though most felt that simulation-based practice would lead to more operative autonomy. There are likely many reasons contributing to this high number. Perhaps the most important is the lack of structured training with a coach, as evidenced in this survey, and the knowledge of how to participate in self-directed learning. Additional reasons include competition for resources for both surgical trainees and faculty, and the availability of simulation task trainers, expendable materials, and established curricula. Future studies into specific barriers to simulation-based practice could highlight additional actionable areas to increase practice rates.

The results of this study suggest several opportunities to enhance laparoscopic training beyond FLS certification. First, surgical residency programs may benefit from developing structured simulation curricula or incorporation of previously validated curricula, like ATLAS, that continue after FLS completion. These curricula should include advanced laparoscopic skills not currently assessed by FLS and be aligned with progressive training milestones.

Second, incorporating regular faculty coaching and feedback into simulation sessions could improve the translation of simulation-based skills into the operating room. Given that most residents in our study reported minimal exposure to coached simulation, broad efforts to train faculty or senior residents as simulation instructors may help address this gap without requiring significant new resources.

Third, residency programs should consider formal orientation to simulation resources early in training. Several respondents reported limited awareness of how or where to practice, indicating that access alone is insufficient without structured guidance and encouragement. Creating simulation task lists tied to clinical rotations could help normalize and prioritize practice.

Finally, as studies like this are conducted, one may ask whether FLS still has a place in training if as many as two thirds of residents can pass without regular practice. The place of FLS and its role for skill development for the trainee may change as surgical training evolves; however, the purpose of FLS remains unchanged. Its importance cannot be understated as it is the only validated tool to ensure minimum knowledge, judgment, and technical skills for laparoscopy. As laparoscopy has become more ubiquitous and may even be considered the standard of care for many cases, one could surmise that many residents will get adequate exposure and develop mastery of these acceptable minimums without the use of simulation-based practice. The Fundamentals of Endoscopic Surgery (FES) skills examination has previously been shown to correlate strongly with clinical experience suggesting that simulation-based practice is not required to achieve a passing score [15]. Future studies may show the same for FLS. Development and proof of mastery of fundamental and advanced laparoscopic skill may need to evolve from a one-time summative exam to include additional scaffolded training tools that are integrated longitudinally throughout residency. Partnerships between national surgical education bodies and individual training programs could foster the implementation of advanced modules that build upon FLS foundations and better align with current operative demands. Such collaboration could additionally help raise awareness of previously developed curricula aimed at addressing these concerns, like ATLAS, and help solve the paradox exposed in this survey that residents believe practice will lead to more operative autonomy but overwhelmingly do not practice.

## Conclusion

The implementation of the FLS program has positively impacted laparoscopic skill acquisition among general surgery residents and has become a key milestone in surgical training. However, our findings suggest that FLS often represents the endpoint, not the beginning, of independent simulation-based practice in laparoscopy. To better align with the increasing complexity and volume of laparoscopic procedures in residency, ongoing adaptation and refinement of the FLS curriculum may be necessary. Encouraging self-directed learning, improving access to simulation resources, integrating structured coaching, and expanding the skill set beyond the five core tasks could enhance the transfer of simulated skills to the operating room. As surgical education evolves, so too must the tools used to train residents, ensuring that certification not only validates competency but also catalyzes continued growth in minimally invasive surgery.
